# Electrophysiological and imaging evidence of sustained inhibition in limbic and frontal networks following deep brain stimulation for treatment refractory obsessive compulsive disorder

**DOI:** 10.1371/journal.pone.0219578

**Published:** 2019-07-19

**Authors:** Hye Ran Park, In Hyang Kim, Hyejin Kang, Kevin W. McCairn, Dong Soo Lee, Bung-Nyun Kim, Dong Gyu Kim, Sun Ha Paek

**Affiliations:** 1 Department of Neurosurgery, Soonchunhyang University Seoul Hospital, Seoul, Korea; 2 Department of Psychiatry, Hanyang University Medical Center, Seoul, Korea; 3 Department of Nuclear Medicine, Seoul National University College of Medicine, Seoul, Korea; 4 Systems Neuroscience Section, Primate Research Institute, Kyoto University, Inuyama, Aichi, Japan; 5 Department of Neurosurgery, Seoul National University College of Medicine, Seoul, Korea; Graduate School of Medical Science, Kyoto Prefectural University of Medicine, JAPAN

## Abstract

Obsessive-compulsive disorder (OCD) is a neuropsychiatric disorder that arises from a complex interaction of environmental and genetic factors. Despite numerous pharmacological and behavioral interventions, approximately 10% of patients remain refractory. High-frequency deep brain stimulation (HF-DBS) has shown promising results for treatment-refractory OCD. We report the follow-up result of up to 6 years of 4 treatment-refractory OCD patients treated by HF-DBS. Targets of stimulation were the anterior limb of the internal capsule (ALIC) in two cases, and the nucleus accumbens (NAc) in the remaining cohort. The clinical profiles were quantified by the Yale-Brown obsessive-compulsive scale (Y-BOCS). Highly significant reductions in Y-BOCS scores were obtained from all patients during the follow-up period. A greater that 90% reduction in Y-BOCS, observed in the most successful case, was achieved with NAc HF-DBS. Y-BOCS scores in the other patients consistently achieved over 50% reductions in OCD symptoms. FDG-PET imaging indicated post-surgical reductions in metabolism, in not only targeted limbic networks, but also other frontal cortical and subcortical regions, suggesting that large-scale network modulation and inhibitions are associated with functional recovery in OCD. This study demonstrates that HF-DBS targeted to the ALIC and NAc is a safe and effective method for ameliorating intractable, treatment-refractory OCD symptoms. The NAc appeared to be the superior target for symptom reduction, and local inhibition of NAc activity and reduced frontal metabolism are key therapeutic indications.

## Introduction

Obsessive-compulsive disorder (OCD) is one of the most disabling psychiatric illnesses; its emergence in patients is associated with intrusive thought and bizarre ritualistic behavioral patterns, which can lead to significant impairments in personal, social, and occupational function for those afflicted. Patients with OCD impulsively and repeatedly think that they should do something regardless of their will. Typical obsessive behaviors include repeated check-ups of door locks, gas burning checks, hand washing, cleaning, walking without step on the line, symmetrically aligning objects, failing to discard objects, giving meaning to sounds, words and numbers, and sexual imagination.

Substantial evidence supports the use of cognitive behavioral approaches and selective serotonin reuptake inhibitors (SSRIs) in the treatment of OCD [1]. Medical treatment using prevents serotonin reabsorption and increases function. Cognitive behavioral therapy has the effect of making negative thoughts recognized as false beliefs through experience. For example, patients with obsession for washing hands may benefit from "exposure reaction prevention method" which prevents them from washing hands after touching dirty clothing or garbage intentionally. By gradually increasing the patient's tolerance, the patient can help control his or her thoughts of compulsion. Most patients will experience at least some symptom relief with these interventions either alone or in combination. However, even after adequate treatment trials, 40% to 60% of patients endorse residual, impairing symptoms after initial treatment [2], prompting the investigation of augmentation strategies, novel pharmacologic agents, and neuromodulatory approaches. Consistent subset of patients, around 10%, remain refractory to any of the above treatment, and further treatment strategies are required [3]. Surgical treatments, including anterior cingulotomy, capsulotomy, subcaudate tractotomy, and limbic leucotomy, have been used for decades for patients with treatment-refractory OCD. The clinical response rate of these surgical treatments was reported to be 50% to 60% after 6–24 months [4].

High-frequency deep brain stimulation (HF-DBS) has emerged as a potential option for treatment-resistant refractory OCD patients. Unlike what is commonly known, the first applications of ablative procedure and chronic stimulation were psychiatric disease, not movement disorders [5]. In 1947, Spiegel and Wycis at Temple University developed a stereotactic apparatus to perform ablative procedures on human beings. In their monumental article, they stated that the apparatus was made for psychosurgery [6]. Delgado and his colleagues, who developed the electrode implantation technique for chronic recording and stimulation, also recorded that it was for 'its possible therapeutic value in psychotic patients’ [7]. Based on the pathophysiology of OCD, several regions with synaptic connectivity in circuits of orbitofrontal cortex (OFC), anterior cingulate cortex (ACC) and striatum has been tried as target of HF-DBS to control the abnormal activity [8]. Targets including the NAc, ALIC, subthalamic nucleus (STN), internal globus pallidus (GPi), and basal nucleus of the stria terminalis (BNST) have been tried, and these trials have shown promising results in refractory OCD patients [9–17]. In meta-analysis, (50–60) % of the patients were treatment responsive, with at least 35% of YBOCS reduction [18, 19]. The reduction rate in DBS severity range from (52%) to (54%) in patients who underwent VC/VS or NAc stimulation, and 41% in those who underwent STN stimulation [20]. The rates of treatment responders after HF-DBS are comparable to those of lesional surgery including capsulotomy and cingulotomy (64%), and ablative technique (56%) [21, 22]. Most patients also regained normal quality of life after HF-DBS [23]. HF-DBS has some disadvantages including high costs, the need for battery change, and some emotional and somatic side-effects, although they are usually mild, transient, and reversible.

Although HF-DBS has shown promising results for treatment-refractory OCD, there remains a degree of heterogeneity in the therapeutic response, due to the relatively small number of cases that have been undertaken and reported. This indicates that much still needs to be understood with respect to the underlying condition and the use of HF-DBS as a treatment option [24]. Here, we present the follow-up result of up to 6 years of 4 treatment-refractory OCD patients who underwent HF-DBS of the NAc and ALIC for OCD based on intraoperative recording and long-term follow up with FDG-PET. We propose a therapeutic mechanism based on suppressed hyperactivity in the frontal and limbic circuits.

## Methods

### Patients

This study was approved by the Institutional Review Board of Seoul National University Hospital (IRB no. H-1609-043-790) and was conducted according to the tenets of the Declaration of Helsinki. No written consent was needed due to the retrospective study design. Potential candidates for OCD treatment with HF-DBS were screened for, and selected, in our OCD clinic at Seoul National University Hospital (SNUH), Korea. In South Korea, DBS was approved for treatment of movement disorders, epilepsy, intractable pain, OCD, and Tourette syndrome by K-FDA. Patients who meet the indication receive insurance benefits. For patient selection, psychiatrists conducted a thorough chart review and assessment of present history, past evaluations/treatments, and family history. The primary diagnosis of OCD was verified according to the Diagnostic and Statistical Manual of Psychiatric Disorders fourth edition (DSM-IV). Inclusion criteria were chronic, severe, and treatment-refractory OCD. Chronic OCD was defined as duration of illness with a minimum of 4 years without any remission periods. OCD symptoms had to be judged objectively to be of a disabling severity, as indicated by the Yale-Brown obsessive-compulsive scale (Y-BOCS ≥ 25). Treatment-refractory OCD was defined as failure to respond to the maximal tolerable dose of at least three different SSRI’s for at least 4 weeks, and with augmentation of at least one atypical antipsychotic drug.

Patients were excluded if they had a current or past psychotic disorder, bipolar disorder, or had clinically significant suicidal tendencies within the past 6 months. Additional exclusion criteria were current or unstably remitted substance abuse or dependence, mental retardation (an intelligence quotient <70), or any medical/neurologic contraindication to HF-DBS surgery. Among the eligible population in our OCD clinic, four patients who met the criteria underwent HF-DBS surgery between May 2005 and November 2012. They gave consent to HF-DBS for OCD, after easily understandable detailed information about the treatment method. Informed consent for surgery was obtained from all patients at the department of neurosurgery of SNUH.

### Surgical procedure

**Fig 1:**
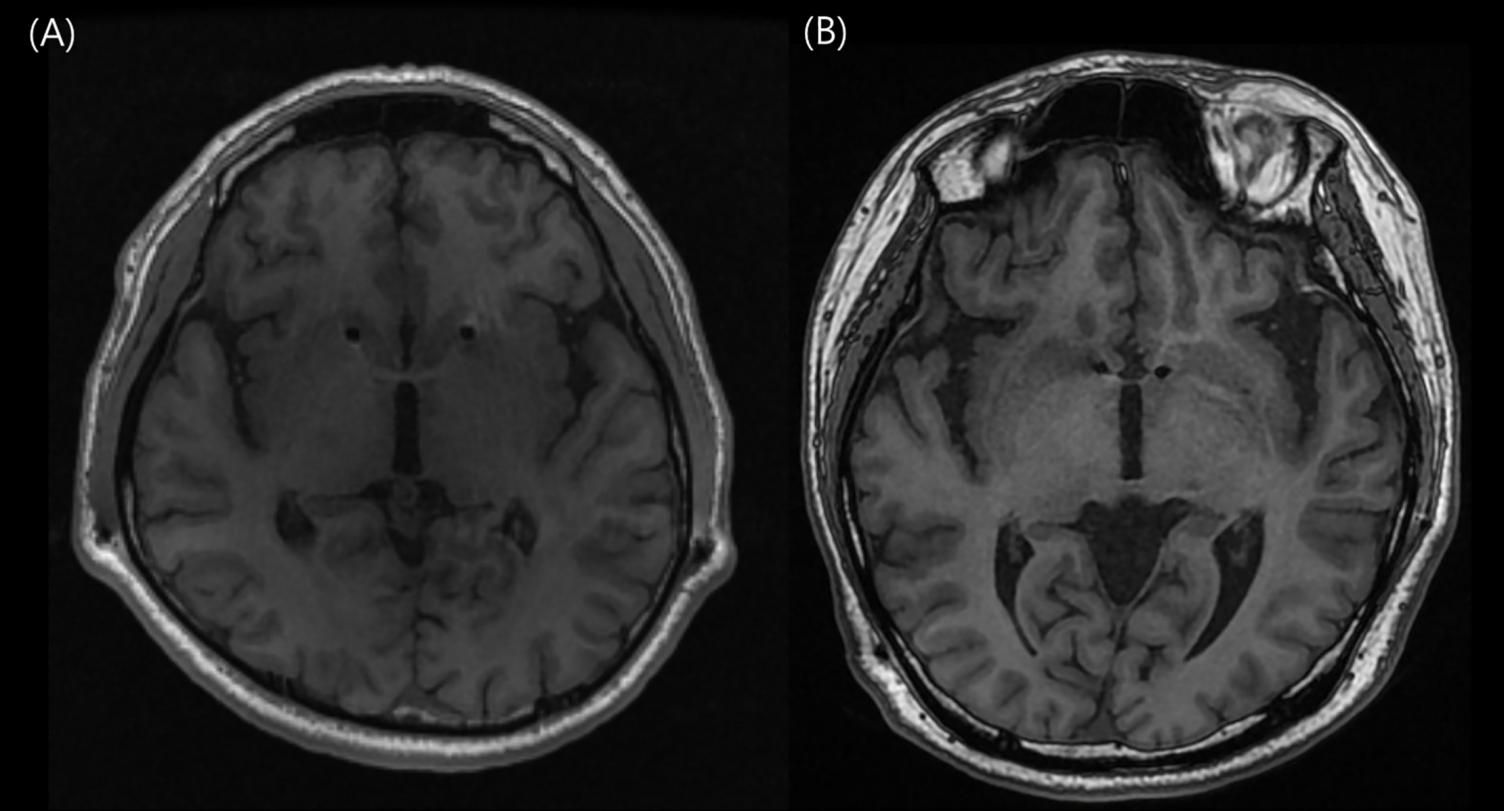
Postoperative magnetic resonance imaging after electrode insertion. (A) Anterior limb of internal capsule (B) Nucleus accumbens.

Quadripolar electrodes (Model 3387 DBS Lead; Medtronic, Minneapolis, MN, USA) were first implanted bilaterally in the anterior limb of the internal capsule (ALIC) of two subjects (Fig 1(A)), and the nucleus accumbens (NAc) in the following two consecutive cases (Fig 1(B)). Under local anesthesia, placement of electrodes was performed utilizing a stereotactic approach with a Leksell frame. MER utilizing a 5-channel discrete trajectories, the so-called “Ben-Gun”, was conducted to map the region of the brain where the HF-DBS probe was to be placed (Differential microTargeting Electrodes, FHC, Chemnitz, Germany;1.5 MO impedance). After changing to general anesthesia, programmable, battery-operated pulse generators were implanted in both subclavicular fossa, and were connected to the electrodes utilizing a tunneling tool for subcutaneous electrical wires.

### Intraoperative HF-DBS stimulation protocol

A feature of this study was that we were able to conduct recordings of neuronal activity in the NAc of diagnosed OCD patients while fully conscious and recorded from the site of stimulation through varying intensities of stimulation. Neural signals were sampled at 40 kHz utilizing a Lead Point MER system (Medtronic, Minneapolis, MN, USA); stimulation was delivered via a DBS lead (DBS 3387, Medtronic, Minneapolis, MN) at a frequency of 130 Hz, giving an interpulse period of 7.6 ms, at an intensity in the range (1–3) mA. Neuronal activity was processed offline; stimulation artifacts induced by pulse delivery, were removed from the high-passed (200 Hz) data stream via a moving average, template subtraction method, similar to our previously published methods. Amplifier saturation prevented analysis of periods approximately (0–3) ms after pulse delivery; however, approximately 4 ms of the interpulse period was amenable to spike sorting at current levels in the 1 mA range. Higher current ranges in the case of NAc electrophysiology were not amenable to spike detection during the stimulation period, due to the low level of spontaneous spiking in this particular brain region and the magnitude of the shock artifact. Comparisons to recorded activity prior to stimulation onset, and its properties as stimulation was stitched off, were used to infer the effect of stimulation on neuronal activity. In the case of stimulation at the 1 mA current level, after removal of the voltage transients caused by the stimulation pulse, individual and multi-unit neural activity was extracted utilizing principal component analysis via Plexon off-line sorter. The time stamps of identified spikes were analyzed and processed in NeuroExplorer and MATLAB environments. We used Perievent analysis aligned to individual stimulation pulses, or to the start (DBS on) or end (DBS off) of DBS periods, to determine the effect of HF-DBS on local spiking activity within the vicinity of the stimulating electrode.

### Postoperative HF-DBS stimulation protocol

Fig 1 shows the HF-DBS stimulation parameters. Postoperative activation of the implanted stimulation occurred approximately one week after surgery was complete in all patients. The initial voltage was 4 V (Case 1), 3 V (Case 2) or 1.5 V (Cases 3, 4), and this voltage was adjusted individually, according to each patient and their clinical response, e.g., improvement/aggravation of OCD symptoms, mood change, and stimulation-related side-effects. Unlike the stimulation voltage, other parameters (pulse width at 60 μsec, frequency at 130 Hz) were fixed.

### Outcome measures

Clinical evaluations were performed with a psychiatric interview at baseline, postoperatively (weekly for a month, and then every two weeks up to 6 months), and at follow-up visits every 1 month. Provisional endpoint of the post-HF-DBS assessment was 24 months. The primary outcome was the mean change of the Y-BOCS total score from baseline, and this was checked at every assessment point. The secondary outcome measures were changes in depression, anxiety, general functioning, and global illness severity which were assessed using the Hamilton depression rating scale (HAM-D), Hamilton anxiety rating scale (HAM-A), Global assessment function scale (GAF), and Clinical global impression (CGI), respectively. These clinical scales were evaluated every month or every other month.

## Results

### Subject clinical profile

#### Subject one

Table 1 summarizes all patient characteristics and treatment responses to HF-DBS. S1, a nineteen-year-old male, was referred to SNUH OCD clinic for severe, refractory OCD accompanied with self-injurious behavior. The initial onset of OCD symptoms started five years previously, and S1 demonstrated a persistent self-checking behavior and a compulsive interrogation of other individuals, with various forms of questions and inquiries. He was particularly capricious and sensitive to events that did not go his way, often self-harming to express his displeasure and anger at a situation, should it prove irritating or frustrating to him. Pharmacological treatment and psychotherapy were unsuccessful, and he had to quit middle school because of his difficulty in maintaining harmonious social relationships with his peers and teachers. Preoperative brain magnetic resonance imaging (MRI) was normal, but ^18^FDG-PET revealed hypometabolism in both frontal lobes, and mild hypometabolism in both parietal and temporal lobes. Electroencephalogram (EEG) showed decreased delta/theta/alpha absolute power and increased beta/high beta relative power in a diffuse central area, suggesting depression. The baseline Y-BOCS was 31.

**Table 1:** Characteristics of all patient and treatment responses to HF-DBS.

Patient No	Subject 1	Subject 2	Subject 3	Subject 4
DBS Year	2005	2010	2012	2012
Sex/Age	M/19	M/39	M/42	F/43
Onset age	14	19	27	38
Disease duration (years)	5	20	15	5
OCD symptom dimension	Aggressive/checking	Aggressive/checking Symmetry/ordering	Aggressive/checking	Hoarding, checking
Comorbidity	Self-injurious behavior, depression	Depression	-	Depression
DBS target	ALIC	ALIC	NAc	NAc
Side	Bilateral	Bilateral	Bilateral	Bilateral
Y-BOCS baseline	31	34	30	26
Y-BOCS postoperative	14	17	2	8
Follow up period (months)	48	72	48	42
% Y-BOCS reduction	55%	50%	93%	69%
Responder (>35%)	+	+	+	+

**Fig 2:**
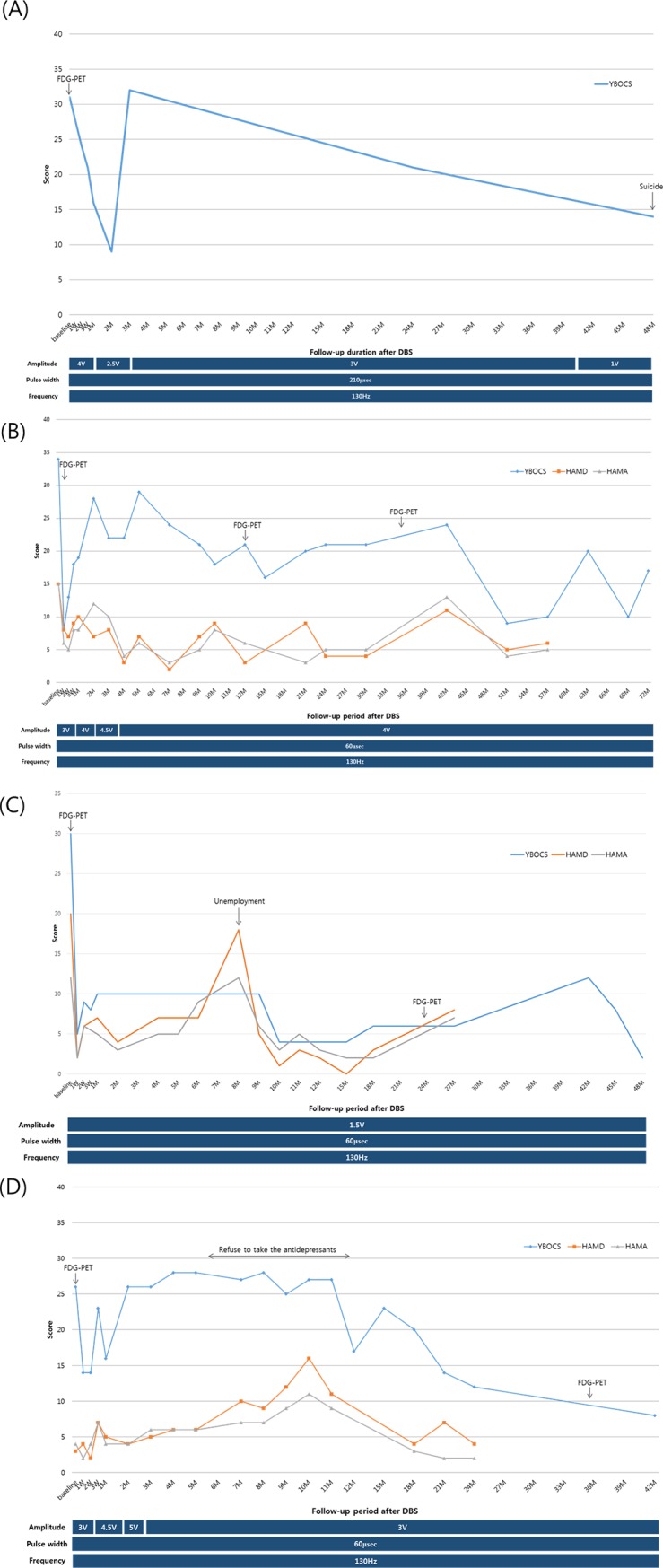
Changes in obsessive-compulsive disorder symptoms of the four patients after deep brain stimulation implantation in bilateral nucleus accumbens. Y-BOCS, Yale-Brown obsessive-compulsive scale; HAM-D, Hamilton depression rating scale; HAM-A, Hamilton anxiety rating scale.

S1 was implanted in the ALIC bilaterally, as described in the methods. Although he experienced transient aggravation of his symptoms at postoperative 3 months, he showed steady reduction in Y-BOCS until postoperative 4 years (Fig 2(A)). The reduction rate of Y-BOCS at the last follow-up point was 55%. He did not show cognitive impairment during the follow-up period, with depression scores being maintained at an acceptable level through closely monitored medication. However, S1 was lost to the study due to death through misadventure; the coroner’s report found no connection between those events and their inclusion in the HF-OCD trial.

#### Subject two

S2 was a thirty-nine year-old male, married, with a twenty-year history of refractory OCD. With an onset at nineteen years old, S2’s symptom was considered refractory but not of a particularly serious nature, generally relating to checking behavior, and an obsession with the number four. Approximately ten years before surgical intervention, his symptoms became aggravated, and started to become an impediment to his occupation as a deliveryman. His compulsion to check the location of objects led to feelings of fear and paranoia if he was unable to structure his daily routine to allow for the behavior that ameliorated his compulsions. This led to a degradation of work performance and conflict with his employers, thus compounding his stress state and his symptoms. Pharmacological treatment and cognitive behavior therapy had proved inadequate interventions for his symptom profile. Neurocognitive testing revealed a moderate range symptoms related to cognition, including deficits related to attention, frontal executive function, and motor control, as well as emotional maladjustment such as depression and anger. The baseline Y-BOCS was 34. Pre-operative electroencephalogram and MRI were negative for any gross abnormalities, and preoperative FDG-PET was unavailable for this subject.

Bilateral ALIC HF-DBS was conducted. The initial stimulation parameters were as follows: amplitude 3 V, pulse width 60 μsec, frequency 130 Hz. Following surgery, the patient showed a dramatic improvement in obsessive thought and compulsive behavior within one month after implantation (Y-BOCS, 8). At postoperative two months, OCD symptoms worsened in spite of the initial positive response to ALIC HF-DBS, and an increase in stimulation amplitude up to 6 V was initiated, in an attempt to restore improved behavioral patterns. Starting from postoperative six months, the patient showed steady improvement in OCD symptoms, with his return to work occurring one year after ALIC implantation, and the patient continued to maintain his improved state. The Y-BOCS measured at postoperative 72 months was 17, and the improvement rate of Y-BOCS was 50% (Fig 2(B)).

**Fig 3:**
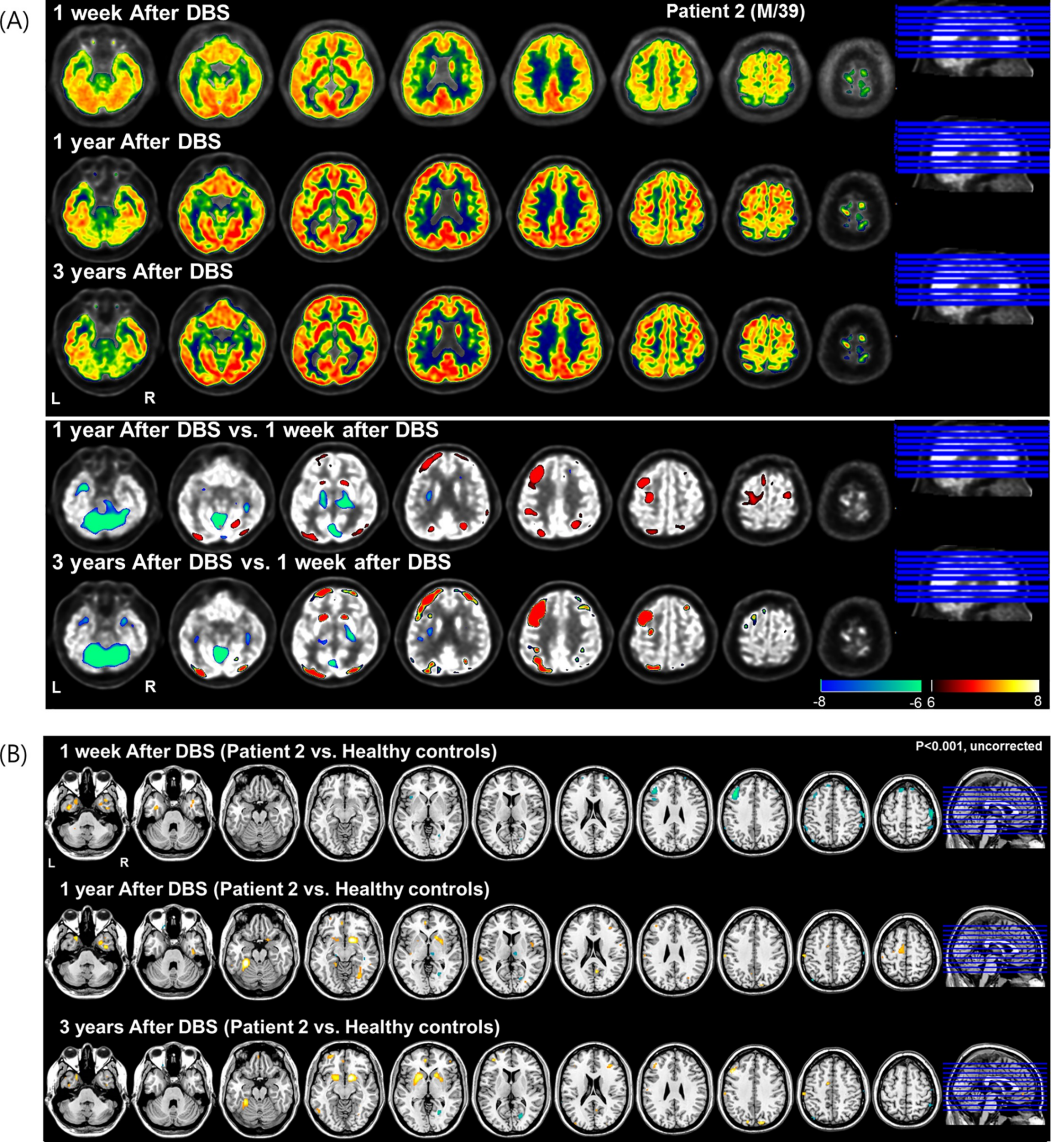
^18^Fluorodeoxyglucose-positron emission tomography (FDG-PET) taken in Subject two at postoperative 1 week, 1 year and 3 years. (A) FDG uptake in each period and subtraction map between pre-and post-operative state in two rows of bottom. The simple subtraction images of normalized FDG uptake between pre and post-operative state showed decreased (winter color map) and increased (hot color map) regional metabolic activity. (B) Relative to healthy controls (N = 24, mean age = 35.2 ± 8.6), the decreased metabolism at postoperative 1week was observed and increased metabolism in bilateral caudate and frontal regions at postoperative 1 year and 3 years was observed (p<0.001, uncorrected for multiple comparisons).

FDG-PET taken at postoperative 1 week revealed decreased metabolic activity in cortical regions, such as the prefrontal and occipital regions (Fig 3(A)). FDG-PET taken at postoperative (1 and 3) years similarly showed increased metabolism in the left prefrontal and bilateral caudate regions compared to 1 week after DBS (Fig 3(B)). Relative to healthy controls, the metabolic activity at postoperative 1 week was decreased in the frontal cortex and focally increased at postoperative (1 and 3) years in the prefrontal and bilateral caudate regions (Fig 3(B))

#### Subject three

S3, a married, forty-two year-old male, had been suffering with OCD for fifteen years prior to surgery. His symptoms started after a severe traffic accident, due to a mechanical failure of the car. Due to this accident and its cause, he became obsessive about the mechanical state of his car, repeatedly checking the car and any small sound that might, in his mind, be indicative of an underlying and potentially dangerous fault. His fear of accidents grew to include generalized anxieties about other hazards, e.g., fires; he was afraid to flip switches in case of short circuits and the potential fire risk that might ensue; and he compulsively checked his vicinity for dangerous objects that could cause bodily harm; and when entering his house, he would constantly dust himself off. Pharmacologic and psychotherapeutic treatments were given over fifteen years without success. Pre-operative brain MRI revealed small focal high signal intensity lesion in T2-weighted images, in the left parietal lobe subcortical area. EEG findings were in the normal range. The baseline Y-BOCS was 30.

Bilateral NAc HF-DBS implantation was performed. Fig 2(C) shows the changes in OCD symptoms in response to NAc HF-DBS. The patient demonstrated bradylalia, and immediately after HF-DBS implantation, seemed to be apathetic. At postoperative one week, it was observed that the dusting-off behavior of this clothes was greatly reduced (Y-BOCS, 5). Although he occasionally had obsessive thoughts, these were not excessively intrusive, and did not affect his daily life. At postoperative 2 months, apathy was improved. OCD symptoms and depression were temporarily increased after unemployment at postoperative 8 months (Y-BOCS, 18), so antidepressant (venlafaxine) was increased to 225 mg. Following surgical intervention, he was able to drive his car with minimal checking, although the behavior did persist slightly. His clinical score for OCD taken at postoperative 48 months was (Y-BOCS, 2), and the improvement rate of Y-BOCS was 93%.

**Fig 4:**
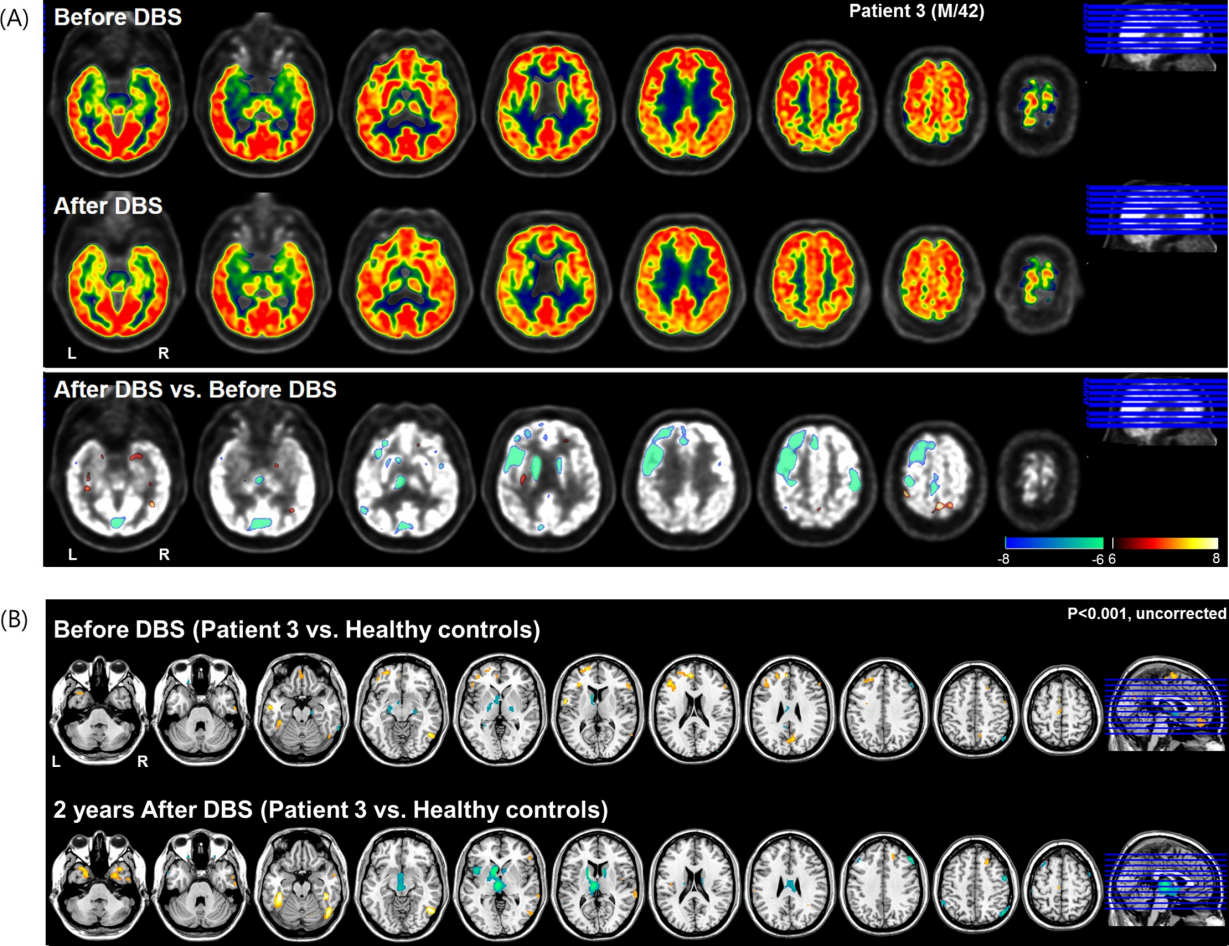
^18^Fluorodeoxyglucose-positron emission tomography (FDG-PET) image taken in Subject three at pre-and postoperative 2 years. (A) Relative to before DBS showed extensively decreased metabolic activity in left frontal including anterior cingulate and bilateral caudate after DBS. (B) This metabolic activity in prefrontal including anterior cingulate regions was hyperactive in before DBS and then at 2 years after DBS, this pattern was recovered or normalized as a normal state (p<0.001, uncorrected for multiple comparisons).

FDG-PET image at postoperative 2 years relative to before DBS showed extensively decreased metabolism in the left frontal area, including the anterior cingulate (Fig 4(A)). This metabolic activity in the prefrontal region including the anterior cingulate regions was hyperactive before DBS, and then at 2 years after DBS, this pattern was recovered or normalized as a normal state (Fig 4(B)).

#### Subject four

S4, a forty-three year-old female, had been suffering with OCD symptoms for five years prior to NAc HF-DBS intervention. The patient had no past or family history of OCD or psychiatric illness, OCD symptoms emerged spontaneously, and centered on kleptomania; S4 would observe objects of interest from people’s houses and gardens and then she would obsess about their physical properties, e.g., color, shape, size, etc., and would lie awake at night planning on how she could acquire them. Acquiring the items would temporarily assuage any obsessive thoughts. Pharmacotherapy and psychotherapy were transiently effective, but her symptoms consistently returned, despite any medication that was trialed. Preoperative brain MRI showed high signal intensities in the T2-weighted image at the bilateral cerebral white matter, suggesting small vessel disease. EEG analysis revealed subtle abnormalities including increased absolute power at all recorded sites, and increased interhemispheric coherence between the frontoparietal areas. The baseline Y-BOCS was 26.

Bilateral NAc HF-DBS implantation was performed. As described in Fig 2D, initially her symptoms were improved after NAc HF-DBS implantation (Y-BOCS, 14). However, obsessive thoughts continued to be a persistent problem for approximately (2–3) hours per day, with the symptoms continuing to get worse (postoperative (4–8) months Y-BOCS, 28). Between postoperative six and eight months, the patient refused to take her prescribed medicines for depression. At postoperative one year, S4 reinitiated antidepressant, and the OCD symptoms, as well as depression and anxiety, decreased (Y-BOCS, 17). The frequency and intensity of compulsive behaviors steadily decreased, and the patient gained new employment. While staying in the dormitory of the new workplace, transient deterioration of compulsive behavior emerged as a compulsion to check the objects in the next room. After leaving the dormitory and living in a rented room, the OCD symptoms and depressive mood were relieved. She could control her impulse to stick to objects. At postoperative three years, her measured symptoms were completely in remission; and at her last assessment postoperative forty-two months, there was continued suppression of OCD symptoms (Y-BOCS, 8), a reduction of 69%.

**Fig 5:**
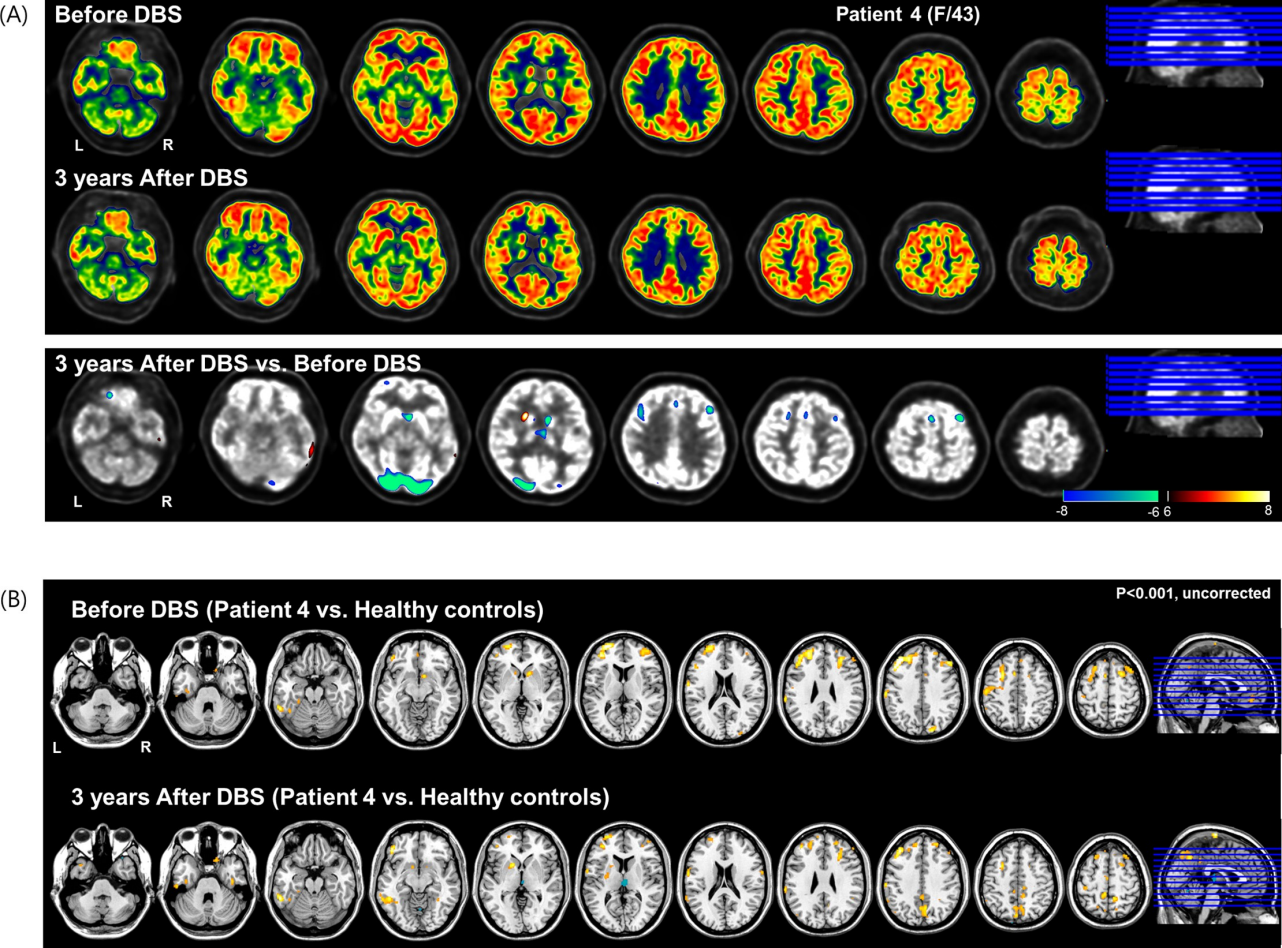
^18^Fluorodeoxyglucose-positron emission tomography (FDG-PET) scan taken before and after NAc DBS in Subject four. (A) After DBS, FDG uptake in bilateral frontal including anterior cingulate and occipital region was decreased compared to before DBS. (B)Relative to healthy controls, the hypermetabolic activity in frontal regions after DBS was less extensive than before DBS (p<0.001, uncorrected for multiple comparisons).

Before DBS, FDG uptake in the bilateral frontal region including the anterior cingulate was increased, compared to not only after DBS, but also healthy control group. After DBS, metabolism in the prefrontal and anterior cingulate was decreased (Fig 5(A)). Relative to healthy controls, the hypermetabolic patterns at postoperative 3 years were less extensive than before DBS (Fig 5(B)).

### Intraoperative NAc electrophysiology

**Fig 6:**
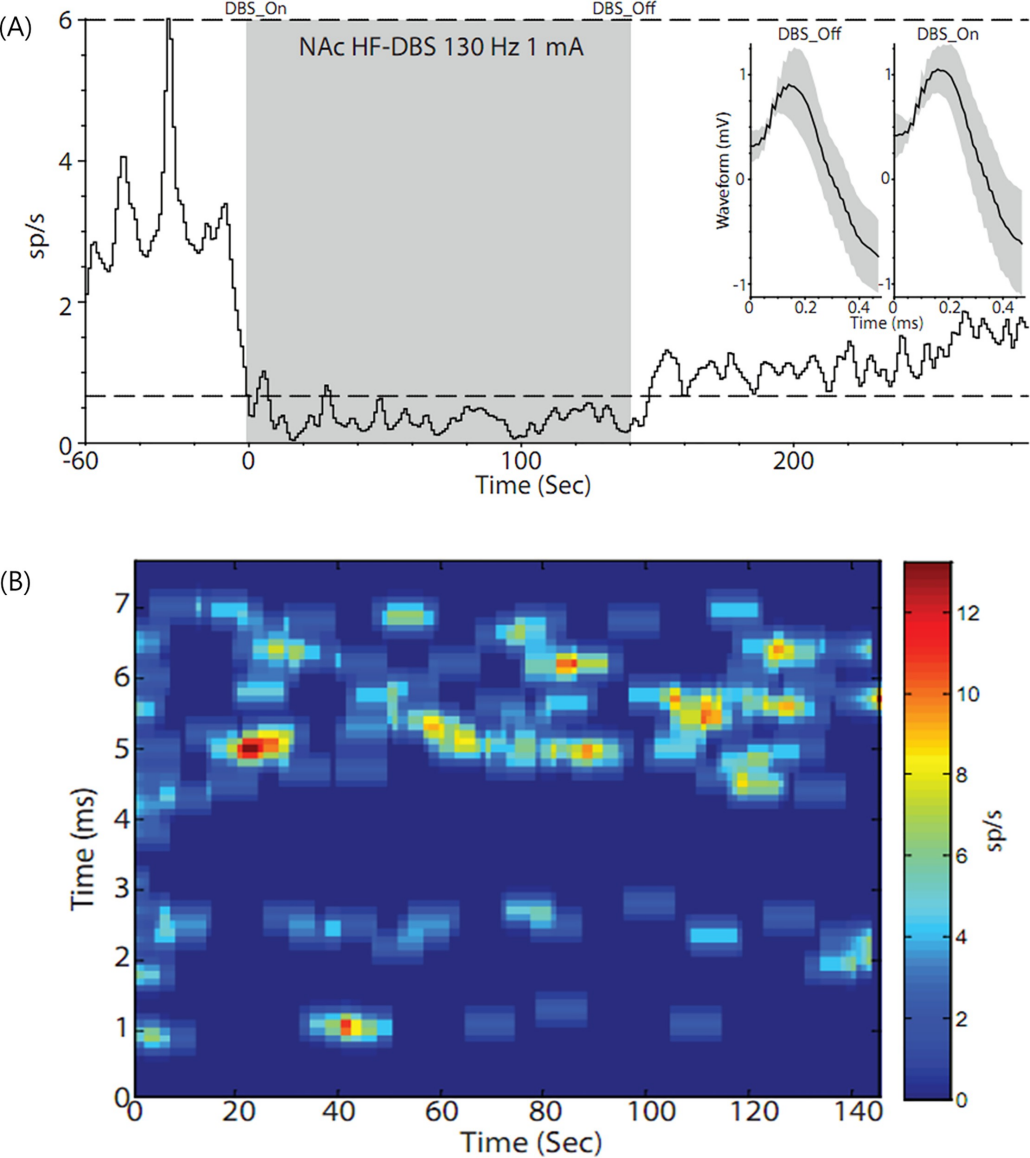
Analysis of the rate firing and neuronal activity. (A) Analysis of the rate firing for one well-isolated unit after extraction of any unwanted voltage transients due to shock artifacts at 1 mA. The figure clearly shows that the mean firing rate (2.93 sp/s SD ± 0.7), drops below the 99% confidence interval calculated from the pre-stimulation period as soon as “DBS_on” is initiated. The units firing rate maintains this reduced activity for the duration of the stimulation period (0.33 sp/s SD ± 0.1 [gray shaded period]). It should also be noted that the mean firing rate slowly rebounds and does not reach the previous levels of activity prior to stimulation (1.05 sp/s SD ± 0.5). (B) Analysis of the neuronal activity during the stimulation period in an attempt to understand the neuronal properties and nature of the inhibition, the tracked unit appears to show a sporadic but ongoing entrainment with the stimulation pulse with action potentials clustering in the 4-7ms range.

During the implantation of the HF-DBS probe into the NAc, we were able to acquire and identify several well-isolated single-units from the immediate vicinity into which the stimulation probe was placed, and a number of multiunit recordings n = 17. This data was acquired across 16 individual recording sites within the NAc. Although the number of single and multi-unit recordings was small, due to the small number of patients who submitted to the protocol, the conditions under which the recording, e.g., time limits with respect to recording electrode placement in the brain, and the inability to manipulate each electrode independently to increase neuronal yield. There was an obvious tendency in the data, that was indicative of a current-related inhibitory response, which persisted even after stimulation ceased. Audibly, this was obvious in the OR environment and instantaneous readouts from the Lead Point display/oscilloscope. Fig 6(A) shows the off-line analyzed rate firing for one well-isolated unit, after extraction of any unwanted voltage transients due to shock artifacts at 1 mA. The figure clearly shows that the mean firing rate (2.93 sp/s SD ± 0.7), drops below the 99% confidence interval calculated from the pre-stimulation period as soon as “DBS_on” is initiated. The units firing rate maintains this reduced activity for the duration of the stimulation period (0.33 sp/s SD ± 0.1 [gray shaded period]). It should also be noted that the mean firing rate slowly rebounds and does not reach the previous levels of activity prior to stimulation (1.05 sp/s SD ± 0.5) (Fig 6(A)). Fig 6(B) shows further analysis of the neuronal activity during the stimulation period in an attempt to understand the neuronal properties and nature of the inhibition. The tracked unit appears to show a sporadic but ongoing entrainment with the stimulation pulse with action potentials clustering in the (4–7) ms range (Fig 6(B)). Although it might sound counterintuitive that temporal entrainment with the stimulation pulse running at 130 Hz, could cause a reduction in the firing rate, this phenomenon is easily explained by the large number of response failures to each individual pulse delivery, approximately (15–17) HF-DBS pulses to one entrained action potential. However, it should be noted that we might be drastically underestimating the actual firing rate during stimulation. This is due to the methods used in this study, methodological constraints make it impossible to identify antidromically driven activity, due to occlusion by the shock artifact. However, we feel that the high response failure and persistent reduction in activity observed in the DBS_Off condition lends credence to an inhibitory mechanism predominating in the striatum in response to NAC-HF-DBS.

**Fig 7:**
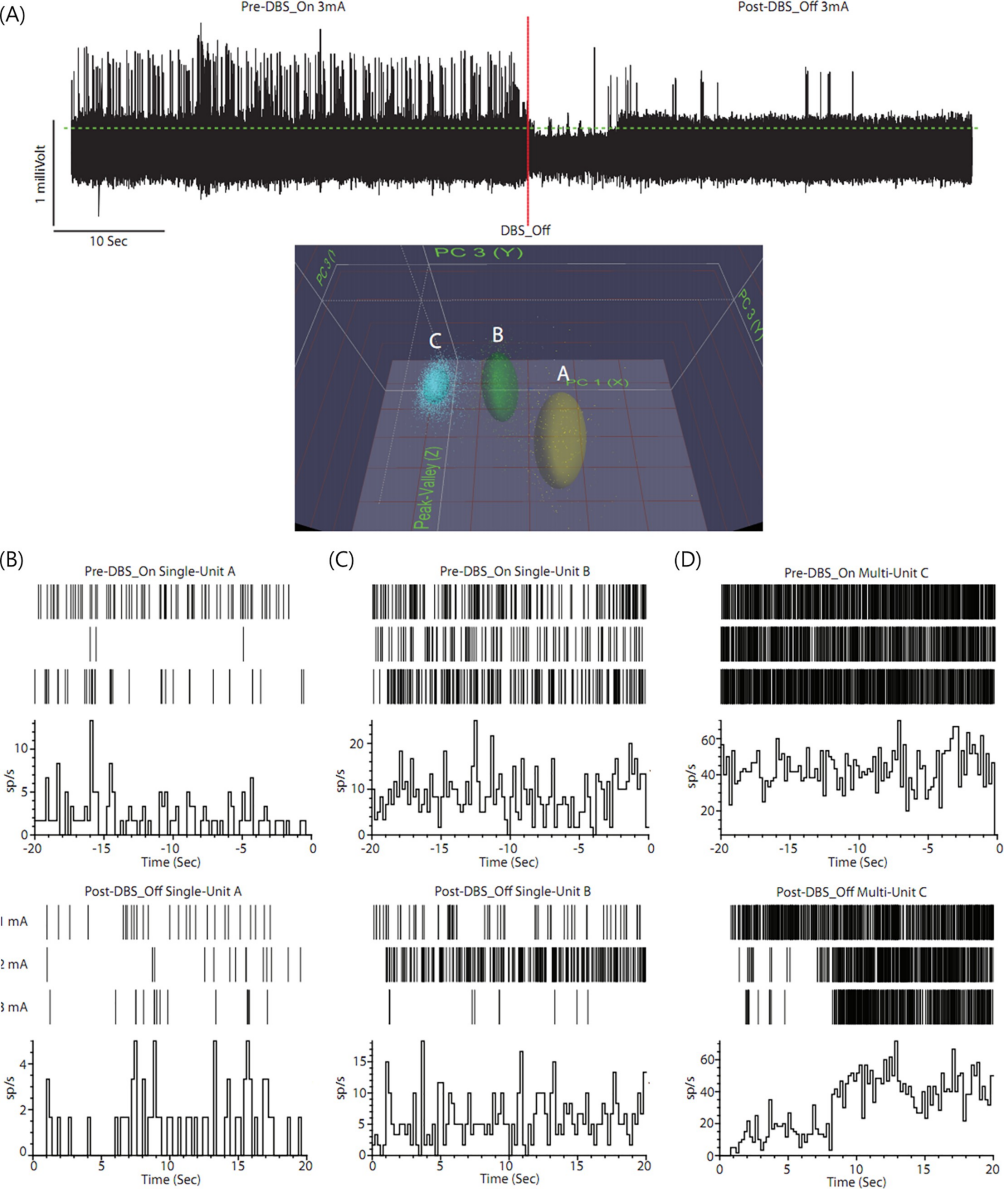
Raw neuronal activity and pre- and post-DBS neuronal activity. (A) Raw neuronal activity in the 40 sec prior to the onset of 3 mA 130 Hz NAc-HF-DBS. The data is clipped to remove the voltage transients from HF-DBS and the trace recommences as the stimulation is terminated. (B)–(D) Pre- and post-DBS neuronal activity in single and multi-unit activity. We identified clear inhibition in single unit activity, but what was also apparent was a reduction in background multiunit activity, that persisted approximately 8–10 seconds in the post stimulation period, perievent histograms aligned to activity prior to “DBS_On” and activity post “DBS_Off”.

Further evidence for inhibitory responses predominating in the NAc can be inferred from the response to higher stimulation currents. Although it was not possible to record in the interpulse period due to distortions from the shock artifact, we observed inhibitory responses when the DBS_Off condition was initiated. Fig 7(A) shows raw neuronal activity in the 40 s prior to the onset of 3 mA 130 Hz NAc-HF-DBS. The data is clipped to remove the voltage transients from HF-DBS, and the trace recommences as the stimulation is terminated. We identified clear inhibition in single unit activity, but what was also apparent was a reduction in background multiunit activity, that persisted approximately (8–10) s in the post stimulation period, and perievent histograms aligned to activity prior to “DBS_On” and activity post “DBS_Off” (Fig 7(B)–7(D)).

### FDG-PET data

Pre- and post-operative FDG-PET was performed in Subjects three and four. In Subject two, postoperative 1 week FDG-PET was performed, instead of preoperative FDG-PET. In Subjects three and four with preoperative FEG-PET, metabolic activity in the bilateral frontal lobe including the anterior cingulate was increased, compared to that of the healthy control group. Postoperative FDG-PET showed decreased metabolism in the bilateral caudate and prefrontal region, compared to preoperative status, and the pre-operative hypermetabolic activity in frontal regions was normalized (S3) and reduced (S4) after DBS. These changes in FDG-PET imaging were correlated with clinical improvement, as described in Fig 2(B)–2(D).

## Discussion

Although the long-term clinical efficacy and safety of HF-DBS has been proven, the therapeutic mechanism of HF-DBS has not yet been fully elucidated. HF-DBS has various effects through orthodromic activation of efferent axons in the neurons within the cortico-basal ganglia loop and antidromic and orthodromic activation of afferent axons. There are three representative hypotheses about the therapeutic mechanism of HF-DBS: inhibition, excitation, and disruption hypothesis [25]. Initially, it was believed that the effect of HF-DBS was to inhibit local neuronal elements, because DBS showed similar therapeutic effects as lesioning procedures. STN HF-DBS showed similar effects for parkinsonian motor symptoms to STN lesioning procedures or STN blockade [9, 26–28]. This inhibition hypothesis fits well with the firing rate and pattern model of movement disorders. HF-DBS reduced the abnormally increased firing rate and abnormal firing patterns of STN and GPi, and alleviated parkinsonian motor symptoms. However, the inhibition hypothesis does not explain the therapeutic effect of GPi DBS on dystonia with low activity. Those who support the excitement hypothesis explain that DBS excite local neuronal elements [29, 30]. Direct evoked spikes of GPi neurons are induced by GPi HF-DBS. STN HF-DBS increased firing of GPi neurons in the PD monkey model, and firing of GPi/GPe and SNr neurons in PD patients through STN-GPi/SNr/GPe projections [31–33]. HF-DBS also antidromically excites afferent axons. The inhibition and excitement hypothesis did not fully explain the therapeutic mechanism of HF-DBS, and disruption hypothesis emerged [25, 34]. The disruption hypothesis is that HF-DBS dissociates the input and output signals, disrupting the flow of abnormal information across the stimulation site. Cortical stimulation results in a triphasic response of GPi neurons leading to early excitement, inhibition, and late excitement [35, 36]. These reactions are mediated by hyperdirect, direct, and indirect pathways, respectively. As a result, the therapeutic effect of HF-DBS is associated with modulating the abnormal activity of the circuits and synaptic connectivity, which is supported by experiments in animal models [23, 37].

The distant HF-DBS effect to abnormal neural connectivity of the cortico-striato-thalamo-cortical circuits explains the similar rates of improvement after stimulation of several brain regions. STN stimulation decreases the metabolism of the OFC and medial prefrontal cortex (PFC) as well as ACC activity [38]. ALIC stimulation is also known to be associated with the metabolism of the OFC [9, 11], subgenual ACC [38], and right dorsolateral PFC [39]. The therapeutic effect of HF-DBS on OCD can also be explained by neurotransmitter. HF-DBS supplements the striatal dysfunction through striatal dopamine increase and reward processing. The aberrant reward processing and phenomenology of OCD are related to abnormal dopaminergic neurotransmittion [40]. Recently, Liebrand et al. found that ventral ALIC DBS for OCD may benefit from medial forebrain bundle (MFB)-specific implantation and emphasized the importance of corticolimbic connections in OCD response to DBS [41]. They hypothesized that treatment response depends on the location of the active DBS contacts with respect to individual white matter bundle trajectories, and performed tractography analysis of two fiber bundles, the anterior thalamic radiation (ATR) and the supero-lateral branch of the MFB. They found that the treatment outcome was better when the active stimulation was located near the ventral ALIC rather than the ATR.

In this study, the two previous patients received ALIC HF-DBS, and the latter two received NAc stimulation. As the role of NAc in the reward circuit gradually became clear, we have changed the target. Since 2012, our group has been using NAc as a stimulation target, instead of ALIC. NAc is a converging area of the reward circuit, and disturbances of the reward circuit are regarded as being related to addiction, depression, and OCD. NAc, part of the ventral striatum, composes the limbic subcircuit of the basal ganglia. NAc integrates the information from the limbic structure and prefrontal cortex, modulates goal-directed behaviors, and composes the main parts of reward circuitry. We also recently reported satisfactory outcome after HF-DBS in an autism patient with self-injurious behavior [42]. Although the number of cases is too small and the outcome cannot be determined by this study alone, the optimal target can be elucidated throughout further basic research on the brain circuits and clinical trials.

The OCD dimension of sexual/religious obsessions and compulsions is correlated with a significantly better response to HF-DBS in meta-analysis studies. Specific functional connectivity pattern in the brain is regarded with this OCD clinical dimension. In analysis of the ventral corticostriatal functional connectivity, patients with sexual/religious obsessions and compulsions had more connectivity between the ventral caudate and anterobasal insular cortex, compared to other symptom dimensions [43]. Since Figee et al. reported that reduction of OCD symptom after HF-DBS is correlated with decrease of excessive frontostriatal connectivity from baseline [44], abnormal insulostriatal connectivity has been suggested to be sensitive to HF-DBS capacity to normalize brain connectivity. Further neuroimaging study is needed for difference of connectivity pattern related to HF-DBS response to each patient of OCD symptom dimension.

Less severe adverse effect is reported after HF-DBS than lesional surgery. About 2.6% of intracranial hemorrhage is reported after HF-DBS [45], while 15.8% after ablative intervention [46]. In our experience, persistent frontal syndrome, cognitive impairment, or personality change did not occur. The most severe stimulation-related adverse effect was the suicide attempt that occurred in the first case. While transient worsening until identification of the optimal stimulation parameter was identified in all patients, it was mild, transient, and reversible.

## Conclusion

HF-DBS for OCD shows favorable outcome in terms of YBOCS reduction from (50–93) %. The therapeutic mechanism of HF-DBS for OCD is related to a reduction of synaptic hyperconnectivity identified with FDG-PET, with an increase in striatal dopamine and improvement of reward processing. In our experience, HF-DBS for treatment-refractory OCD has shown adequate effect on clinical symptoms, and acceptable safety. HF-DBS may be an effective treatment for refractory OCD.

### Limitation

The interpretation of these findings has the limit of insufficient data of neurocognitive scale due to the retrospective design. In addition, some patients did not receive imaging and clinical follow-up as scheduled.
